# Writing the Engram: Epigenetic Mechanisms of Memory Allocation

**DOI:** 10.1111/jnc.70328

**Published:** 2025-12-30

**Authors:** Isabella Tarulli, Rebecca Toscano‐Rivalta, Lisa Watt, Johannes Gräff

**Affiliations:** ^1^ Laboratory of Neuroepigenetics, Brain Mind Institute, School of Life Sciences Ecole Polytechnique Fédérale Lausanne (EPFL) Lausanne Switzerland; ^2^ Synapsy Research Center for Neuroscience and Mental Health Research Ecole Polytechnique Fédérale Lausanne (EPFL) Lausanne Switzerland

**Keywords:** allocation, chromatin, engram, epigenetic, excitability, memory

## Abstract

Memory allocation, the selective recruitment of neurons into ensembles that encode, store and retrieve experience, so‐called engram cells, designates the initial step of every memory's formation. Historically thought to be governed primarily by intrinsic neuronal excitability, recent studies highlight a critical role for transcriptional and epigenetic heterogeneity in biasing neuronal engram inclusion. Here, we review mechanisms that influence this process, including CREB‐mediated excitability, transcriptional priming and epigenetic modulation, and emphasise the surprisingly understudied link of how electrical properties and the epigenetic landscape converge to shape allocation. We then describe emerging methodologies for the manipulation and interrogation of these processes that will be crucial for disentangling not only local intracellular dynamics, but also their propagation across distributed brain networks. Doing so will prove instrumental to assess the possibility that several cognitive dysfunctions—that display aberrant excitability and epigenetic changes—may arise from memory misallocation, stressing the translational potential of this work. Lastly, beyond the role of the intrinsic neuronal properties, we discuss underexplored physiological influences, including metabolic state, hormonal signalling, sleep, gut‐brain communication, and the potential contribution from other cell types such as astrocytes and interneurons that may shape engram selection. By integrating molecular, cellular and systems perspectives, with a sharpened emphasis on the importance of epigenetic mechanisms, we suggest that understanding allocation may benefit from a holistic viewpoint beyond the current excitability‐focused and neuron‐centric point of view.

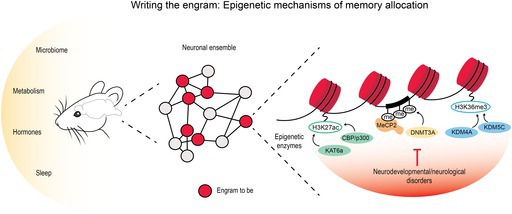

## Graphical abstract

Memory allocation refers to the selective recruitment of neurons to engram cells upon memory formation. While previous studies have documented the importance of intrinsic excitability and the transcription factor CREB on memory allocation, epigenetic mechanisms have remained poorly understood. Here, we summarise recent discoveries epigenetic mechanisms in memory allocation, hypothesise how neurological diseases might be rooted in memory mis‐allocation and outline how various physiological influences can shape engram selection. Finally, we review existing and emerging methodologies in the field, providing a resource for future interrogation of this first step in the life of a memory.

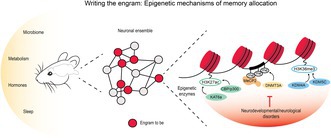

**Abbreviations**
5C‐seq: chromosome‐conformation‐capture carbon‐copy sequencing
AD: Alzheimer's disease
ADHD: Attention deficit hyperactivity disorder
aFC: auditory fear conditioning
ARTHS: Arboleda‐Tham Syndrome
CBP: CREB binding protein
CFC: contextual fear conditioning
ChIP: chromatin immunoprecipitation
ChIP‐qPCR: quantitative polymerase chain reaction
ChR2: receptor channelrhodopsin‐2
CNO: clozapine‐N‐oxide
CRANE: Cross‐Regional Afferent Network of Memory Ensembles
CREBS133A: dominant negative mutant
CREBWT: endogenous CREB
CUT&RUN: cleavage under targets and release using nuclease
CUT&Tag: cleavage under targets and tagmentation
DEGs: differentially expressed genes
DG: dentate gyrus
DNMT3A: DNA methyltransferase 3A
Dox: doxycycline
DREADD: designer receptor exclusively activated by designer drug
e‐LTP: early long‐term potentiation
ERP: event related potential
FACS: fluorescent activated cell sorting
FANS: fluorescent activated nuclei sorting
FISH: fluorescence in situ hybridisation
FRET: Förster resonance energy transfer
FUS‐BBBO: focused ultrasound blood–brain barrier opening
GAM: genome architecture mapping
HATs: histone acetyltransferases
HDAC: histone deacetylase
HP1‐β: heterochromatin protein 1
HPC: hippocampus
HSV: herpes simplex virus
IE: intrinsic excitability
IEGs: immediate early genes
IMRs: intermediate methylated regions
KAT5: lysine acetyltransferase 5
KAT6A: lysine acetyltransferase 6A
KDM4A: lysine‐specific demethylase 4a
LA: lateral amygdala
LADL: light activated dynamic looping
l‐LTP: late long‐term potentiation
MRXSCJ: mental retardation X‐linked syndromic Claes‐Jensen type
MWM: Morris water maze
ORCA: optical reconstruction of chromatin architecture
PKA: protein kinase A
PSDs: post‐synaptic densities
PYP: photoactive yellow protein
RTS: Rubinstein‐Taybi syndrome
RTT: Rett syndrome
SAHA: suberoylanilide hydroxamic acid
scRNAseq: single‐cell RNA sequencing
snmC‐seq2: single‐nucleus methylome sequencing
snRRBS: reduced representation bisulfite sequencing
SST+: somatostatin positive
TBRS: Tatton‐Brown‐Rahman syndrome
TCS: triclosan
TMS: transcranial magnetic stimulation
TRAP: targeted recombination in active populations
TRE: tetracycline response element
TRPV1: transient receptor potential cation channel V1
tTA: tetracycline transactivator
VTA: ventral tagmental area

## 1 Introduction

Memory can be defined as an experience‐dependent alteration in behaviour that persists beyond the environmental stimuli that produced it. Physiologically speaking, memories are stored in a subset of brain cells, nowadays referred to as engram cells. To define a specific memory, the engram ensemble undergoes a multi‐staged process, that comprises memory allocation, encoding, consolidation and recall.

While the molecular and cellular mechanisms underlying memory encoding (i.e., the process of converting the information into a format that can be stored in our memory), consolidation (i.e., the process of keeping the information in our memory to be accessible at a later time), and recall (i.e., the process of retrieving the same information from our memory) have been extensively studied over the past decades, the molecular basis of memory allocation, the process by which specific neurons are selected to encode a given memory, forming the engram ensemble, has only recently started to gain significant attention.

The first studies on the cellular properties of memory allocation have centralised on the role of intrinsic excitability (IE) along with expression levels of the transcription factor CREB (cAMP response element‐binding protein) and immediate early genes (IEGs) as core determining elements for engram trace recruitment. However, upstream factors governing these fluctuations remained, until recently, unknown. Emerging evidence now implicates epigenetic heterogeneity, defined as differences in chromatin state not pertaining to DNA sequence modification, as an intrinsic factor that biases neurons towards recruitment into the memory trace. These discoveries rest upon recent advances in engram labeling and single‐cell epigenomic profiling technologies that have enabled the characterisation of how gene expression and chromatin dynamics, including histone modifications, DNA methylation and chromatin remodelling, contribute to the regulation of different phases of memory after allocation (Halder et al. 2016; Jaeger et al. 2018; Fernandez‐Albert et al. 2019; Gulmez‐Karaca et al. 2020; Marco et al. 2020; Chen et al. 2020).

This chapter begins with a brief summary of the well‐established roles of CREB and IEGs in memory allocation before integrating recent findings that highlight the importance of transcriptional and chromatin changes as key upstream mechanisms guiding the preferential recruitment of neurons into memory ensembles.

### 1.1 CREB, Excitability and Neuronal Competition

The first molecular insights into memory allocation originated in the observations that CREB upregulation via stereotaxically injected herpes simplex virus (HSV) in a subset of lateral amygdala (LA) neurons biased their recruitment into the engram ensemble (Han et al. 2007). Interestingly, some years before, overexpression of CREB was already found to be highly involved in enhancing memory formation, namely by increasing long‐term memory expression after massed fear training (Josselyn et al. 2001). This illustrated the importance of CREB activity in different phases of memory, but brought forth questions regarding downstream mechanisms at play.

In response, subsequent studies showed that neurons with higher IE, likely as a consequence of elevated CREB levels, exhibited enhanced synaptic strength compared to neighbouring neurons, which increased their inclusion probability into the memory ensemble (Zhou et al. 2009; Yiu et al. 2014). These findings gave rise to the concept of a competitive nature of allocation to a given engram, whereby neurons with higher excitability ‘win’ the competition to encode the memory (Frankland and Josselyn 2014). Importantly, CREB also plays a functional role in subsequent memory expression, as CREB‐overexpressing neurons are preferentially reactivated at recall (Han et al. 2009). On the contrary, inactivation of these molecularly manipulated neurons by reversible allostatin‐driven potassium channel activation prior to retrieval leads to memory impairments (Zhou et al. 2009).

These studies confirmed that, once biased towards allocation, these neurons become functionally essential components of the engram, as their activity is both necessary and sufficient for memory expression. However, what determines why some neurons exhibit higher CREB expression or excitability than others, especially when receiving similar inputs, remains poorly understood. While this excitability‐based mechanism of allocation is recapitulated under physiological conditions, with natural fluctuations of IE determining which neurons are selected to encode the memory (Gouty‐Colomer et al. 2016), the timescales of excitability plasticity might differ from those of CREB changes, and thus whether endogenous fluctuations in CREB dictate allocation into the engram remains to be determined. Likewise, how CREB‐independent intracellular pathways modulate excitability and how this regulates excitability‐related gene programs is equally under‐examined and will be important to study in future research.

## 2 Epigenetic Regulation of Engram Allocation

### 2.1 Epigenetic Plasticity: A Pre‐Learning Priming Code for Memory Recruitment

Although it is well established that synaptic activity initiates transcriptional cascades that are modulated by epigenetic mechanisms (Levenson and Sweatt 2005), this concept has traditionally been associated with memory formation, consolidation and recall, including in engram cells (Halder et al. 2016; Jaeger et al. 2018; Fernandez‐Albert et al. 2019; Marco et al. 2020; Chen et al. 2020). Interestingly, many CREB target genes regulate chromatin remodelling and histone acetylation, suggesting that CREB's role in pre‐learning neuronal eligibility may itself be modulated by the epigenetic state of a neuron. Recent findings support this view, implicating epigenetic cell‐to‐cell variability which ultimately influences neuronal functioning (Odell et al. 2020; Guo et al. 2024; Santoni et al. 2024). As we will discuss, these variations appear to establish a primed state in a subset of neurons, predisposing them for incorporation into the engram ensemble, suggesting that even within the same developmentally defined cell type, not all neurons are equally suited for encoding and storing memories.

### 2.2 Histone Acetylation and Chromatin Accessibility

Direct evidence linking histone acetylation levels to IE and engram allocation was provided for the first time by Santoni et al. (2024). Endogenously fluctuating levels of histone acetylation, specifically acetylation of lysine 27 on histone 3 (H3K27ac), was found to determine memory allocation, indicating that chromatin plasticity and epigenetic state act as a pre‐learning priming factor that determines which neurons are eligible for information encoding. In more detail, principal neurons in the LA displayed naturally occurring heterogeneity in chromatin compaction by means of heterochromatin protein 1 (HP1‐β), a marker of transcriptionally silent heterochromatin. Similarly, heterogeneity in H3K27ac was observed across the LA, but was specifically elevated in neurons expressing the IEG cFos following memory formation. Correspondingly, overexpression of histone acetyltransferases (HATs), i.e., the enzymes that catalyse the addition of acetyl groups to histones, such as CREB binding protein (CBP) or lysine acetyltransferase 5 (KAT5), increased H3K27ac and induced a preferential recruitment of these neurons into the engram. Conversely, their downregulation prevented neurons from being recruited, indicating necessity and sufficiency of chromatin plasticity in terms of histone acetylation in biasing allocation.

Subsequent concurrent analyses of chromatin accessibility and gene expression revealed that histone hyperacetylation increased chromatin openness and upregulated genes linked to neuronal excitability, structural remodelling and synaptic plasticity. These epigenetically primed neurons were further morphologically characterised by increased spine density and, as demonstrated by ex vivo patch‐clamp recordings, exhibited increased IE. Behaviourally, CBP and KAT5‐injected mice displayed a stronger memory following contextual fear conditioning (CFC) up to 8 days following encoding, indicating improved memory retention mediated by the allocated neurons. Concordantly, optogenetic silencing of the HAT‐overexpressing neurons abolished memory recall, demonstrating a causal link between chromatin state and memory expression. Lastly, Förster resonance energy transfer (FRET) tools and calcium imaging further demonstrated an endogenously occurring cell‐autonomous, real‐time correlation between histone acetylation and higher IE, thereby solidifying the role of chromatin plasticity as a pre‐learning determinant of the physiological properties that steer allocation.

### 2.3 DNA Methylation

While Santoni et al. focused on histone modifications, another epigenetic modality priming genetically identical cells to support memory formation was described by Odell et al. (2020): DNA methylation. In this study, the focus was on DNA methyltransferase 3A (DNMT3A), which catalyses the addition of methyl groups to cytosine bases. This enzyme is essential for the developmental emergence of intermediate methylated regions (IMRs), which define bistable epialleles (methylated or unmethylated) that regulate neuron‐specific excitability and recruitment. In the dentate gyrus (DG) of the hippocampus (HPC), conditional knockout of DNMT3A led to hypomethylation of IMRs at the level of CpG sites (loci where cytosine is followed by guanine). Neuronal activity was also reduced as measured by calcium activity, albeit without impaired behaviour during a novel object recognition task. Conversely, DNMT3A overexpression increased methylation at IMRs, elevated neuronal excitability as measured by whole cell patch‐clamp slice recording, and enhanced the probability of recruitment into the encoding ensemble, without altering the overall engram size.

When comparing recruited (FOS positive) and non‐recruited (FOS negative) neurons 1 h after novel environment exposure, single nucleus reduced representation bisulfite sequencing (snRRBS) revealed increased methylation at IMRs in recruited DG neurons. This is in support of the view that DNA methylation acts as a molecular switch, biasing not only neuronal fate, but also neuronal activation. Interestingly, targeted methylation editing at specific epialleles directly affected neuronal excitability and ultimately recruitment in the engram network, suggesting that the epigenetic makeup of only few genomic sites might ultimately be capable of driving memory allocation.

### 2.4 Histone Methylation

Whereas histone hyperacetylation and DNA hypermethylation serve as permissive epigenetic priming events, repressive epigenetic marks can also negatively bias memory allocation. Recently identified in a CRISPR‐Cas9 knockout screen during fear memory formation, histone lysine‐specific demethylase 4a (KDM4A) was found to function as a key negative regulator of memory allocation (Guo et al. 2024). This enzyme demethylates tri‐methyl residues on lysine 9 and 36 of histone 3—marks associated with gene repression and activation, respectively (Couture et al. 2007; Tsukada et al. 2006). Of these two modifications, knockdown of KDM4A in DG neurons led to an increase in H3K36me3 only, as well as increased neuronal activation and likelihood of recruitment to the engram, again without expanding the overall engram size. Downstream mechanisms were revealed by bulk RNA sequencing showing upregulation of the calcium influx and learning‐associated gene *Trpm7* (Transient Receptor Potential Cation Channel Subfamily M Member 7). This was a consequence of reducing KDM4A which increased H3K36me3 levels, permitting activity‐dependent burst transcription and thus preparation of synaptic proteins for memory encoding. Functionally, loss of KDM4A induced an enlargement of mossy fibre boutons, thereby linking changes in chromatin remodelling, presynaptic plasticity and circuit maturation for memory allocation.

### 2.5 An Emerging Model of Epigenetically Driven Memory Allocation

**FIGURE 1:**
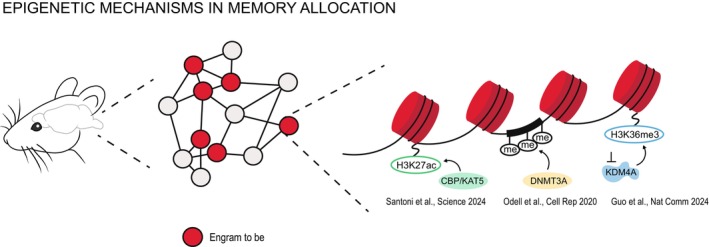
The formation of an engram involves the recruitment, among all the neurons involved in a circuit (grey and red), of specific neurons (red) based on their chromatin conformation during a specific event. Different levels of epigenetic regulation of chromatin plasticity, highlighted here, accompany the allocation of memories during learning. (1) CBP/KAT5 increase H3K27ac level, which leads to increased chromatin accessibility, enhanced transcription of synaptic‐related genes and higher neuronal excitability. (2) DNMT3A enhances the level of methylation at the level of specific CpG islands, thereby increasing transcription and neuronal excitability. (3) The removal of the epigenetic repressor KDM4A allows for the increase of H3K36me3 and transcription of genes necessary for memory encoding.

Together these studies converge on a shared mechanism, according to which, prior to learning, epigenetic heterogeneity serves as a molecular code for memory allocation (Figure 1), through histone hyperacetylation and increased chromatin accessibility (Santoni et al. 2024), transcriptionally permissive DNA methylation patterns at bistable loci (Odell et al. 2020) or through reduced epigenetic repression mediated by histone hypomethylation to brake engram inclusion (Guo et al. 2024). A more transcriptionally permissive chromatin state therefore primes neurons for neuronal excitability and transcriptional readiness, which is then actively solidified by neuronal activity‐induced opening of chromatin and acetylation at key genes (IEGs, synaptic genes), finally leading to structural and synaptic remodelling necessary to consolidate the allocation into long‐term memory traces.

## 3 Allocation in Pathology

Despite the well‐established importance of memory allocation in proper cognitive functioning, the potential for misallocation in pathological conditions remains unexplored. In addition, it is unknown whether these processes could be epigenetically mediated. However, there is substantial reasoning to support this possibility, as we will outline in this chapter. Indeed, several neurological disorders harbour mutations in memory allocation‐relevant genes such as CREB, CBP/p300 and DNA methylation enzymes, which argues that memory impairments associated with these disorders may, at least partially, stem from aberrant memory allocation mechanisms.

### 3.1 CREB Alterations—The Case of Schizophrenia

Schizophrenia affects approximately 24 million people worldwide, with diagnosis usually occurring during late teens for men and late twenties for women (World Health Organisation 2022). This disorder impacts how individuals perceive and interact with the world around them, with symptoms ranging in type and severity, but broadly consisting of distortions in thought, perception and behaviour (Mayo Clinic 2024). The root cause of schizophrenia remains unknown, but has been proposed to involve a mix of genetics, environmental factors and neurotransmitter signalling. For the latter, schizophrenia patients show enhanced D2 dopamine receptors (D2R)‐mediated dopaminergic activity but a deficit in D1R‐mediated dopaminergic activity (Toda and Abi‐Dargham 2007).

Interestingly, CREB has been proposed to be a convergent dopaminergic signalling protein in schizophrenia (Dudman et al. 2003; Balu and Coyle 2018; Kawanishi et al. 1999; Zheng et al. 2012). Collectively, these studies suggest that in physiological conditions CREB gets activated either by dopamine receptors through Protein Kinase A (PKA) or Akt‐mediated phosphorylation, which in turn promotes the expression of target genes such as Brain Derived Neurotrophic Factor (BDNF). In pathological conditions, CREB phosphorylation is lowered and thus promotes a dampened expression of BDNF and other target genes.

Considering the link between the allocation‐associated CREB and BDNF, it is reasonable to propose a link between the memory impairments observed in schizophrenia and the alterations in how memories are allocated, which could be readily tested in various mouse models of the disease.

### 3.2 Acetylation‐Related Pathologies

#### 3.2.1 Rubinstein‐Taybi Syndrome: CBP and p300 Mutations

Rubinstein‐Taybi syndrome (RTS) is a rare neurodevelopmental disease occurring in 1/100000 to 1/125000 live births (Milani et al. 2015). Caused by variants in either of two genes encoding for the proteins CBP and p300, it is characterised by intellectual disability, well‐defined facial features, distal limb anomalies and atypical growth, among numerous other symptoms (Lacombe et al. 2024).

Interestingly, RTS‐mimicking CBP+/− mice display both memory deficits and lower levels of the transcriptionally permissive histone variant 2B (H2B) acetylation (Alarcón et al. 2004). Moreover, CBP+/− mice have normal early long‐term potentiation (e‐LTP) but a deficiency in late LTP (l‐LTP; Alarcón et al. 2004). While e‐LTP relies solely on local modifications of already present synaptic players, l‐LTP depends on the induction of specific gene expression patterns, as it requires the synthesis of new mRNA and proteins (Martin et al. 2000). Correspondingly, treatment of CBP+/− mice with the histone deacetylase (HDAC) inhibitor suberoylanilide hydroxamic acid (SAHA) not only reverted their histone acetylation deficits, but also restored l‐LTP and their memory capacities (Alarcón et al. 2004). Taken together, these findings suggest disrupted memory allocation as a functional link between histone hypoacetylation and impaired memory formation in CBP+/− mice, and by extension, in RTS, although this remains to be tested.

#### 3.2.2 Arboleda‐Tham Syndrome: KAT6A Mutations

Similar to RTS, another cognitive disorder related to HAT mutations is Arboleda‐Tham Syndrome (ARTHS). This ultra‐rare genetic disorder is caused by a de novo heterozygous nonsense mutation in lysine acetyltransferase 6A (KAT6A; Zeng et al. 2022). ARTHS was first reported in 2015 and since then only 300 cases have been diagnosed (Yu et al. 2025). This disorder features intellectual disability as well as developmental and speech delays (Singh et al. 2023).

The role that KAT6A might play in the intellectual deficits in ARTHS has recently been confirmed using a novel mutant mouse model of the disorder (Liu et al. 2024). KAT6A mutant mice displayed deficits in recognition and spatial memory as well as in an auditory fear conditioning. Notably, deletion of KAT6A in hippocampal pyramidal neurons recapitulated these impairments, indicating that loss of KAT6A specifically in excitatory neurons is sufficient to disrupt memory function (Liu et al. 2024). Such neurological deficits were caused by a significant decrease in hippocampal CA3 spine density and reduced levels of the signalling protein Wnt. Interestingly, defects in Wnt signalling have been linked to dampened neuronal IE as well as to the regulation of spine morphogenesis (Oliva and Inestrosa 2015; Ramos‐Fernández et al. 2021). Therefore, ARTHS—caused by KAT6A/WNT signalling deficits—may also represent a disorder of malfunctioning memory allocation.

### 3.3 DNA Methylation‐Related Pathologies

#### 3.3.1 Rett Syndrome: MeCP2 Mutations

The X‐linked disease Rett syndrome (RTT) is caused by mutations of Methyl‐CpG‐binding protein 2 (MeCP2). RTT is considered the second leading cause of mental retardation in women, with a pooled prevalence estimate of 7 in 100 000 females (Sweatt 2009; Petriti et al. 2023). The hallmarks of RTT are continuous and stereotypical hand movements, decreased growth including microcephaly, abnormal respiration, gait ataxia, autism and seizures alongside learning disabilities and cognitive deficits (Sweatt 2009).

RTT‐associated cognitive deficits are primarily attributed to mutations of MeCP2, a model of RTT. In line with this, Hao et al. (2015) reported that MeCP2+/− mice exhibited impairments throughout the learning phase of the Morris Water Maze (MWM), which were rescued by deep brain stimulation targeting synaptic plasticity and hippocampal neurogenesis. Consistent with these findings, Moretti et al. (2006) showed that RTT mice displayed not only spatial but also fear learning deficits. The same study also identified synaptic alterations in the CA1 region, in the form of reduced post‐synaptic densities (PSDs) and altered basal synaptic functioning (paired‐pulse facilitation, LTP LTD). Using another MeCP2 heterozygous mutant mouse line, RTT mice also showed impaired learning during a novel object location test (Li et al. 2017).

One potential molecular underpinning of such synaptic defects could be that, when mutated, MeCP2 can no longer interact with its effectors (both of transcriptional activating and repressing nature), among which CREB presents itself as a possible candidate (Chahrour et al. 2008). Crucially, as we have seen, CREB enhances the IE of neurons, thus giving them a competitive advantage for inclusion in the memory ensemble (Zhou et al. 2009; Sano et al. 2014; Lavi et al. 2023), thereby positing RTT as a potential disorder of memory allocation. In line with this, He et al. (2022) found that in the CA1 of RTT mice there is an imbalance in the ratio of excitation‐to‐inhibition (E/I) due to a lower recruitment of a subset of somatostatin interneurons, called OLM (oriens‐lacunosum moleculare) cells. As a result, the allocated engram size is larger in RTT mice than in WT and these mice also perform worse in a CFC paradigm.

#### 3.3.2 Tatton‐Brown‐Rahman Syndrome: DNMT3A Mutation

Tatton‐Brown‐Rahman syndrome (TBRS) is caused by *de novo* mutations of DNA methyltransferase 3A (DNMT3A). It is characterised by obesity, different degrees of intellectual disability, joint hypermobility, hypotonia, behavioural/psychiatric issues and seizures. Individuals with TBRS also have subtle dysmorphic features and have an increased risk of developing acute myeloid leukaemia (Ostrowski and Tatton‐Brown 2022). Its prevalence is not known yet as only 90 cases have been reported so far (Ostrowski and Tatton‐Brown 2022).

TBRS‐associated cognitive impairments are primarily attributed to mutations of DNMT3A. In line with this, Odell et al. (2020) elegantly showed that increasing the levels of DNA methylation and the proportion of the methylated epialleles at bistable regions enhances intrinsic excitability. This in turn increases the chances of neurons to be allocated into a new memory. Because in TBRS, the mutations of DNMT3A are affecting the normal functioning of the enzyme, it is possible that the cause of memory deficits observed in patients are caused by alteration in neuronal IE, in turn linked to memory allocation.

### 3.4 Histone Methylation‐Related Pathologies

#### 3.4.1 Attention Deficit Hyperactivity Disorder: KDM4A Mutations

Attention deficit hyperactivity disorder (ADHD) covers two broad diagnostic criteria: symptoms relating to hyperactivity, and those concerned with inattention (Tripp and Wickens 2009). Its global prevalence is estimated at 8% for children and adolescents and 3.1% for adults, depending on the diagnostic methods (Ayano et al. 2023).

From a meta‐analysis carried out by the ADHD working group of the Psychiatric Genomics Consortium, in which over 20 000 diagnosed individuals were compared with over 35 000 controls, *Kdm4a* was identified as a risk gene for ADHD (Demontis et al. 2018). KDM4A has direct implications in memory allocation, as its hippocampal knockdown leads to increased neuronal activity and allocation to the engram ensemble (Guo et al. 2024). ADHD patients generally present reduced levels of intracortical inhibition measured by transcranial magnetic stimulation (TMS), compared to healthy controls (Dutra et al. 2016). To this end, analysis of event related potential (ERP) spiking in the adult brain of ADHD patients found that the ERP phase most associated with working memory showed reduced neuronal firing (Kim et al. 2014). This evidence, along with general deficits in memory shown in both human patients and rodent models (Luo et al. 2019; Tian et al. 2024), compellingly suggests memory allocation deficits to contribute to ADHD.

#### 3.4.2 Mental Retardation X‐Linked Syndromic Claes‐Jensen Type: KDM5C Mutations

Mental retardation X‐linked syndromic Claes‐Jensen type (MRXSCJ) is a neurodevelopmental disorder, in which patients exhibit impaired intellectual development, microcephaly, hyperreflexia, and aggression (Guerra et al. 2020). The pathological underpinnings of MRXSCJ lie in nonsense and missense mutations in *KDM5C*. This enzyme is recruited to demethylate H3K4 upon recognition of di‐ and tri‐methylation and generally acts as a transcriptional repressor. Mouse models of MRXSCJ replicate the abnormalities observed in patients: KDM5C KO mice froze less following both auditory fear conditioning (aFC) and CFC paradigms as compared to control animals. Furthermore, KDM5C mice had an increased latency to reach a hidden platform in the MWM, suggesting that KDM5C plays a role in memory acquisition. In line with this, these mice also display defects in dendritic arborisation and spine morphology (Iwase et al. 2016).

The memory deficits in MRXSCJ likely arise from the altered H3K4me profile that both patients and mouse models display. For instance, the loss of KDM5C enhanced the basal expression levels of activity‐regulated genes such as *Arc* (activity‐regulated cytoskeleton‐associated protein), *cFos* and *Npas4* (Scandaglia et al. 2017). Thus, KDM5C knockout, contrary to intuition, dampened the inducibility of these target genes rather than enhancing transcriptional modulation. This could well lead to mis‐allocation of such cells to a new ensemble during memory encoding, but remains to be confirmed.

### 3.5 Alzheimer's Disease

Although Alzheimer's disease (AD) has been traditionally linked to memory loss through the degeneration of neurons and exhaustion of glial cells, a growing body of research in the past decade has begun to explore the possibility that memories are not actually lost, but instead become inaccessible under natural conditions. These inaccessible memory ensembles are more commonly called silent engrams (Roy et al. 2016; Perusini et al. 2017). Intriguingly, in a mouse model of AD known as 5xFAD, conditional deletion of the cell death regulator *Bax* led to improvements in a novel object location paradigm and in CFC (Mishra et al. 2022). This enhanced performance was linked to an increased number of newly formed neurons allocated to the memory ensemble, rescuing the normally reduced immature neuron recruitment and memory function in this mouse model. Moreover, the increase in newborn neurons restored dendritic spine density both in immature and mature engram neurons and made AD engram neurons transcriptionally similar to wild‐type (WT) engram neurons.

Overall, this suggests another possible hypothesis for the loss of memories occurring in AD, namely that fewer neurons are allocated to the engram population. In turn, this may be linked to the loss of dendritic spines, which could decrease the likelihood that the neurons possess the functional features required for allocation to the engram population. In other words, there may already be deficits at the allocation phase responsible for the reduced capability to form new memories. However, to confirm this hypothesis further studies are required.

## 4 Future Perspective—Beyond Epigenetic Plasticity and Neuronal Excitability

In addition to epigenetic plasticity as a novel contributor in determining neuronal recruitment into engram traces, other factors may also play a significant, yet unexplored, role because they directly impact the epigenome. These factors include cellular metabolism, other cell types or peripheral influences such as hormone signalling, sleep and the gut microbiome.

### 4.1 Metabolism as Factor Upstream of Epigenetics

Epigenetic mechanisms are tightly coupled to the metabolic state of a cell, implicating metabolic factors as key modulators of gene expression and, consequently, neuronal function (Mews et al. 2019). To name a few, fed states are associated with high levels of acetyl‐CoA which promotes HAT activity, thereby increasing chromatin accessibility and transcriptional activation (Bradshaw 2021). Similarly, HDAC activity is mediated by the balance of oxidised and reduced nicotinamide adenine dinucleotide (NAD+/NADH), with low NAD+ levels impairing HDAC function, resulting in altered gene expression (Vogelauer et al. 2012). Other metabolites involved in epigenetic regulation include alpha‐ketoglutarate and late triclosan (TCS) intermediates such as succinate and fumarate, which influence histone and DNA demethylase activity (Pizzorusso and Tognini 2020; Tarhonskaya et al. 2017), as well as lactate which directly mediates gene transcription via histone lactylation (Zhang et al. 2019; Merkuri et al. 2024).

Beyond such metabolites, even neurotransmitters themselves can shape the epigenetic landscape. Serotonin, for example, contributes to histone H3 serotonylation in serotonergic neurons, a modification associated with transcriptional activation of cell type‐specific genes (Farrelly et al. 2019). Similarly, histone dopaminylation has been shown to play a pivotal role in driving the aberrant transcriptional plasticity of the ventral tagmental area (VTA) dopamine neurons in response to cocaine exposure (Lepack et al. 2020). Since dopamine metabolism has been linked to CREB activity (see Chapter 3), dopamine signalling itself is likely regulating memory allocation.

### 4.2 Other Cell Types Such as Astrocytes and Inhibitory Neurons

While the majority of studies on memory allocation have focused on excitatory neurons, other cell types such as astrocytes and inhibitory interneurons are very likely to also partake in this process.

For example, a seminal study demonstrated that disruption of glycogen metabolism in hippocampal astrocytes results in an amnesic phenotype for long‐term memory recall (Suzuki et al. 2011). Interestingly, this process was found to depend on astrocyte‐to‐neuron lactate transfer which, when impaired, led to a reduction in *Arc* and *CREB* after learning, suggesting a possible link between astrocytic metabolic function and neuronal allocation. Moreover, there is direct evidence of astrocytic involvement in engram dynamics. In the ventral hippocampus, engram neurons and surrounding astrocytes were found to engage in coordinated calcium waves marking the beginning and end of memory recall, which was recapitulated by optogenetic activation of the excitatory neurons in the dorsal hippocampus (Suthard et al. 2024). Since there is also a direct, real‐time relationship between calcium fluctuations and histone acetylation (Santoni et al. 2024), this raises the intriguing possibility that the synchrony between astrocytes and neurons could be implicated in memory allocation.

Another cell type to consider is the one of interneurons, of which somatostatin positive (SST+) interneurons were found to be implicated in memory encoding by regulating the size of the neuronal memory ensemble in the DG by laterally inhibiting surrounding granular cells (Stefanelli et al. 2016). Interestingly, epigenetic effects within this interneuron population are known to be important during development, where knockout of DNMT1 in SST+ neurons results in aberrant cortical organisation, increased seizure severity and frequent repetitive motor behaviours, which are often linked to neurological and neuropsychiatric diseases (Reichard et al. 2025). This raises the intriguing possibility that a developmentally controlled interneuronal epigenetic regulation of network excitability may gate the allocation of excitatory neurons into engrams by altering the pool of neurons available for recruitment during learning, as shown by Odell et al. (2020).

### 4.3 Peripheral Influences: Hormones, Sleep and the Gut Microbiome

Hormones such as oestrogen or steroids are powerful modulators of neuronal excitability and epigenetic mechanisms, and thereby destined to also influence memory allocation. For the former, oestrogen fluctuations during the estrous cycle have been shown to modulate neuronal signalling in VTA dopaminergic neurons. In female mice, neuronal excitability was found to only change only during the estrus phase following the oestrogen peak (Shanley et al. 2023). In turn, such fluctuating excitability could have epigenetic underpinnings as the chromatin landscape greatly fluctuates over the estrus cycle (Jaric et al. 2019).

For the latter, stress hormone fluctuations were recently found to conceivably bias which neurons are allocated to the engram ensemble during differing physiological states (Lesuis et al. 2021, 2025). Furthermore, glucocorticoid receptor (GR; a target of cortisol/corticosterone) signalling is known to also have prominent roles on the epigenetic regulation of learning and memory (Trollope et al. 2012, 2017; Wu et al. 2017; Paes et al. 2024). Thus, while acute stress leads to improvements in cognitive performance by deposition of the transcription‐promoting marks H3S10p and H3K14ac at the *cFos* and *Egr1* loci (Chandramohan et al. 2008; Gutièrrez‐Mecinas et al. 2011), chronic glucocorticoid exposure leads to impairment in cognitive processes (De Kloet et al. 2005; McEwen 2001) among others via GR binding at the *Hdac2* (*Histone deacetylase 2*) locus (Gräff et al. 2012) – known to negatively impact learning and memory (Guan et al. 2009). Intriguingly, GRs themselves are epigenetically regulated, namely by experiences such as early life stress (Weaver et al. 2004), suggesting that developmentally‐environmentally induced hormonal signalling may also predispose the process of memory allocation.

Beyond hormones, sleep also has an impact on neuronal excitability, although the effects are not clear. Focusing on cortical excitability, several sleep deprivation studies have been conducted in humans which point to an increase in IE measured as global synchronised activity (Cirelli 2017), while other brain areas such as the hippocampus in rats demonstrated decreased neuronal excitability upon sleep deprivation (McDermott et al. 2003). On the epigenetic level, DNA methylation is known to fluctuate in a diurnal pattern, which is impacted by sleep deprivation (Ämmälä et al. 2025; Powell and LaSalle 2015), suggesting that sleep, or a lack thereof, could influence the neuronal epigenetic landscape available for allocation, potentially biasing which neurons become engrams.

Lastly, there are several lines of research pointing to a positive influence of the gut microbiome on neuronal excitability (Darch and McCafferty 2022) and thus memory allocation. For example, one study reported an increase in hippocampal excitability with the administration of the probiotic *E. faecium* and the prebiotic agave inulin, which resulted in improved cognitive performance. Of note, these effects were found to be mediated by butyrate production—a short chain fatty acid that acts as a histone deacetylase inhibitor, which relaxes the chromatin structure and results in increased gene transcription (Romo‐Araiza et al. 2018) – suggesting an epigenetic mechanism as a mediator of peripheral gut microbiome influences.

Overall, these peripheral influences, ranging from hormonal cycles and stress to microbiome composition and sleep, exert demonstrable effects on epigenetic plasticity and neuronal excitability but future studies are needed to causally link these peripheral inputs to intracellular allocation dynamics. In the final chapter, we outline by which tools such research could be conducted.

## 5 Toolbox of Current and Future Research

**FIGURE 2:**
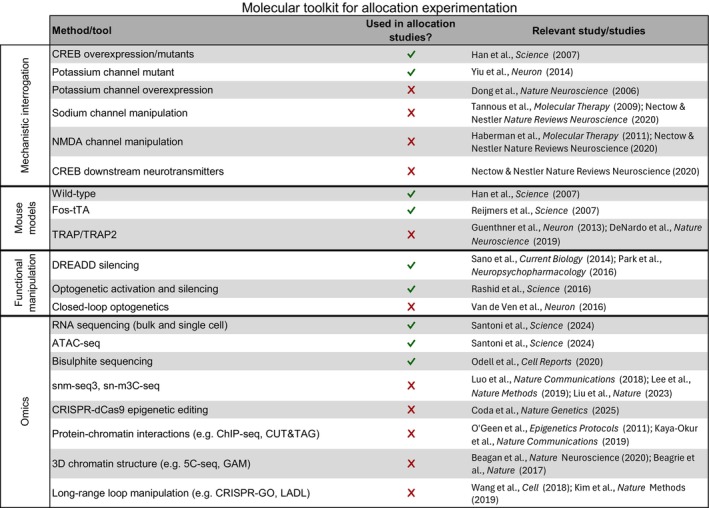
Overview of technologies that have and have not been used in the study of memory allocation with relevant studies for each technology.

In this section, we give an overview of tools that have already been used in the field of memory allocation and explore emerging tools that could be applied to further foster its understanding (Figure 2).

### 5.1 Mechanistic Interrogation

A long‐standing and robust method for investigating the roles of particular proteins in memory research is through expression manipulation. Thus, the pioneering paper from Han et al. (2007) discovered the role of CREB in allocation by overexpressing either the endogenous CREB (CREBWT) or a dominant negative mutant (CREBS133A) in the LA of wild‐type mice. CREBS133A contains a point mutation that renders it phospho‐inactivatable thereby inhibiting its endogenous function (Barrot et al. 2002). These sequences were packaged alongside a GFP reporter inside a replication defective herpes simplex viral vector and stereotaxically injected at a low titre to ensure sparse infectivity. Next, engrams were identified post‐mortem using Arc protein levels to determine allocation bias in GFP‐positive neurons (Han et al. 2007). This method of manipulation can provide many different options for the investigation of specific proteins in memory, including temporal‐specificity. For example, a paper from Ali et al. (2015) described an analogous dominant negative CREB (which binds to endogenous CREB to prevent DNA binding) that is fused to the N‐terminal sequence of photoactive yellow protein (PYP) derived from the bacterium *Halorhodospira halophila* , undergoing conformational changes in the presence of blue light (Ali et al. 2015). In a subsequent study, this new characteristic of the protein was used to toggle the inhibitory properties of the dominant negative CREB on endogenous CREB in the context of allocation (Park et al. 2020).

Despite extensive investigation into the role of CREB in allocation, virtually all mechanistic engram studies have focused on this transcription factor, forgoing investigations into alternative transcription factors or even downstream proteins such as synaptic proteins. Notably, McClung and Nestler (2003) identified several proteins which were upregulated by CREB, but downregulated with its dominant negative mutant. Among these were N‐methyl‐D‐aspartate (NMDA), potassium and sodium channels (Dong et al. 2006; Marie et al. 2005; McClung and Nestler 2003; Zhou et al. 2009), as well as BDNF and the axon growth gene stathmin‐like 2, all of which influence excitability, synaptic plasticity or LTP (Beilharz et al. 1998; Patterson et al. 2001; Schmitt et al. 2009). These proteins have yet to be studied specifically in the context of memory allocation, but can readily be targeted using viral vectors, which allow for cell‐type as well as temporal specificity through the virus type, tailored promoters and post‐transcriptional silencing (Kelly and Russell 2009; Kuroda et al. 2008; Nectow and Nestler 2020; Wang et al. 2023). Moreover, recent advancements in gene editing and transcriptional control, such as using CRISPR‐Cas9 (Ran et al. 2015; Zhuo et al. 2021), or its enzymatically dead version (dCas9; Coda et al. 2025; Gilbert et al. 2013, 2014; Perez‐Pinera et al. 2013), provide enhanced precision for dissecting the molecular mechanisms underlying memory allocation. These dCas9 tools have also been modulated to allow for light‐sensitive control—further increasing the temporal acuity available (Muench et al. 2025).

With IE largely underlying the role of CREB overexpression in allocation (Dong et al. 2006; Zhou et al. 2009), this electrical property of the cell can also be targeted in a similar manner. This has for example been investigated with a dominant negative version of the voltage‐dependent potassium channel subunit KCNQ2 (Yiu et al. 2014). Much like for CREB, the KCNQ2 mutant reduced the activity of the endogenous version, decreasing the flux of potassium and further cementing the role of IE for memory allocation.

Importantly, CREB and excitability‐mediated memory allocation has thus far only been investigated in a limited number of brain regions (Lavi et al. 2023; Park et al. 2020; Park et al. 2016; Zhou et al. 2009), while other engram‐forming regions and those involved in memory encoding and retrieval more generally speaking remain largely unexplored (Dixsaut and Gräff 2021; Guskjolen and Cembrowski 2023; Roy et al. 2022; Tonegawa et al. 2015). Brain‐wide studies would enable the generation of a larger‐scale “allocation map” permitting insights into which brain areas are preferentially engaged during engram formation and how they interact with each other across broader neuronal networks. To achieve this goal, the continued development of systemic‐oriented approaches of intravenous adeno associated virus (AAV) delivery (Challis et al. 2019) might prove particularly useful by enabling a non‐invasive approach to central nervous system targeting.

### 5.2 Mouse Models

As memory allocation concerns the formation of putative engram cells, transgenic mouse lines designed for engram research offer a valuable tool for investigating allocated neurons. These models revolve around the use of IEGs as proxies for neuronal activity (DeNardo and Luo 2017; Greenberg and Ziff 1984; Guzowski et al. 2005; Link et al. 1995; Morgan and Curran 1989). One widely used example is the TetTag mouse line (Reijmers et al. 2007), in which the promoter of the IEG *cFos* drives the expression of a tetracycline transactivator (tTA). In the absence of the drug doxycycline (Dox), tTA binds to the tetracycline response element (TRE) promoter allowing for the expression of a reporter or effector gene placed downstream of it. The expression of this gene—deliverable via a transgenic mouse model or viral vectors—can thus be temporally controlled by Dox administration.

The *cFos*‐tTA system has been widely used for engram identification. However, this system can present limitations when investigating longer‐lasting changes. This is due to tTA driving the expression of a transgene under the TRE promoter only during the tagging window. Consequently, the expression of the transgene may decrease over time due to natural protein turnover, limiting the use of this mouse line when investigating long‐term or persistent molecular/structural changes (Reijmers and Mayford 2009; Kitamura et al. 2017). To overcome this limitation, other mouse lines such as the Targeted Recombination in Active Populations (TRAP; Denny et al. 2014; Guenthner et al. 2013) and the improved TRAP2 (DeNardo et al. 2019) have been created. These transgenic mice also utilise IEGs (in this case *cFos* or *Arc*), but to drive the expression of the tamoxifen‐dependent Cre recombinase CreERT2. Thus, either a fluorescent marker (e.g., tdTomato) or any other floxed protein—again introduced via either transgenic or viral delivery—can be expressed with temporal specificity in putative engram cells (DeNardo and Luo 2017). Due to permanent transgene recombination, the TRAP system allows for long‐lasting labelling.

### 5.3 Functional Manipulations

With these engram techniques, neuronal activity of allocated engram cells can be readily altered through the use of chemogenetic and optogenetic approaches. The most commonly used chemogenetic approach consists of designer receptor exclusively activated by designer drug (DREADD) systems (Alexander et al. 2009). DREADDs are those of the hM1‐5D family of engineered muscarinic acetylcholine receptors which respond to clozapine‐N‐oxide (CNO) instead of acetylcholine. These receptors can be delivered virally, leading to inward rectifying calcium currents upon CNO injection, resulting in neuron silencing (Pei et al. 2008). In the context of neuronal allocation, DREADDs have been paired with CREB overexpression to silence neurons which were biased for engram allocation (Park et al. 2016; Sano et al. 2014).

Chemogenetic manipulation can also be achieved using several other established tools: by the Daun02 inactivation method, in which administration of Daun02 to the TetTag mouse‐line leads to neuronal silencing in beta‐galactosidase‐expressing neurons (Cruz et al. 2013; Engeln et al. 2016; Khalaf et al. 2018; Koya et al. 2009, 2012; Reijmers et al. 2007); by transient receptor potential cation channel V1 (TRPV1) overexpression, a depolarising vanilloid receptor naturally expressed by peripheral nociceptive neurons agonised with the infusion of capsaicin (Kim et al. 2014; Zemelman et al. 2003); or by the *Drosophila melanogaster* allostatin receptor, which leads to neuronal silencing upon the infusion of allostatin (Czajkowski et al. 2014; Lechner et al. 2002).

While DREADDs have proven to be highly effective for neuronal modulation, their temporal resolution is limited due to the pharmacokinetics of clozapine‐N‐oxide (CNO), peaking around 45–50 min post‐injection and returning to baseline after around 9 h (Alexander et al. 2009). A significantly more refined temporal window can be achieved using optogenetics (Kim et al. 2017), which involve light‐sensitive opsins for rapid and reversible control of neuronal activity. The original commonly used activator opsin is the algae‐derived inward cation transmembrane receptor channelrhodopsin‐2 (ChR2) responding to blue light (Park et al. 2016; Zhang et al. 2006). In contrast to activation, inactivation has also been used in allocation studies with the expression of the yellow light‐sensitive chloride pump inhibitory opsin, halorhodopsin, derived from the archaeon *Halobacterium* (Gradinaru et al. 2010; Lau et al. 2020; Rashid et al. 2016; Santoni et al. 2024).

Although DREADDs and optogenetics have readily been used for the study of memory allocation (and in other domains of memory study), there are continued developments of tools that could provide additional or new avenues for research. One example is ATAC (acoustically targeted chemogenetics), which relies on systemically delivered AAVs expressing a DREADD with focused ultrasound blood–brain barrier opening (FUS‐BBBO) used to direct the expression of the AAVs to specific areas of the brain. Upon administration of CNO, pharmacological activation or inhibition can be achieved (Szablowski et al. 2018). When tested in mice, this system was able to successfully increase the activity of hippocampal neurons, measured as the percentage of FOS positive neurons in those also positive for the DREADD (Szablowski et al. 2018).

Finally, another promising direction for functional manipulations involves the use of closed‐loop optogenetic systems by which light laser control can be coupled to the freezing of the animal in real time, increasing temporal precision to further investigate the causality of allocated neurons in memory formation. This could involve the use of neural activity‐coupled monitoring optogenetic stimulation or inhibition (Silva et al. 2021; van de Ven et al. 2016; Zhang et al. 2018). Such approaches could be performed either during endogenous learning conditions or when memory allocation has been otherwise favoured or tempered with, to provide near real‐time resolution on the sufficiency or necessity of these neurons in memory allocation and beyond.

### 5.4 Molecular Interrogation

The molecular underpinnings of to‐be‐allocated neurons remains a sparsely explored avenue of investigation within the field. The major challenge here lies with the identification of these neurons prior to allocation. Current evidence and tools link increased IE and chromatin dynamics to preferential recruitment (Santoni et al. 2024; Zhou et al. 2009), thus artificial manipulation of either factor can yield cells with increased probability of recruitment. However, this pool of cells still remains heterogenous. Thus, future studies should aim to understand whether there are certain factors that endogenously drive allocation. For example, a new technology known as Ca^2+^ split‐TurboID (CaST) allows for the backwards tagging of calcium activity (Zhang et al. 2024). Here, the biotinylation enzyme, TurboID, is split over two AAV constructs, one containing calcium calmodulin and one its binding element. In the presence of calcium, the two constructs bind and bring together the split‐TurboID elements. Increasing biotin levels via intrapleural injection then permits the biotinylation of nearby proteins. Thus, temporal tagging of cells with high calcium levels is achieved (Zhang et al. 2024). Paired with cFos staining following fear conditioning, this technique would offer a correlation between the intracellular calcium levels and cellular recruitment for memory allocation.

With methods such as artificial biasing or CaST, holistic insights into the molecular underpinnings of to‐be‐allocated neurons can be gained using ‐omics‐based tools. The foundational level of analysis in this field is transcriptomics, where the upregulation or downregulation of RNA is used to infer the activation or repression of molecular pathways at specific timepoints or with experimental conditions. Transcriptomic profiling gives a read‐out of differentially expressed genes (DEGs), offering a snapshot window into the molecular landscapes associated with neuronal allocation. This can be performed in bulk (bulk RNAseq) where RNA is extracted from a pool of cells, e.g., allocated engram cells, providing an averaged transcriptional profile (Cho et al. 2016; Marco et al. 2020; Rao‐Ruiz et al. 2019). Here, IEG‐driven transgenic approaches (see Section 5.2) such as the *cFos*‐tTA × TRE‐H2B‐GFP (Tayler et al. 2013) or *Arc‐*Cre × SUN1‐GFP TRAP mouse line (Herzog et al. 2020; Mo et al. 2015) can be used to label engram neurons for subsequent isolation after a performed memory paradigm. One such dissociation method is fluorescent activated cell sorting (FACS; Guez‐Barber et al. 2012; Vembadi et al. 2019). However, FACS has limitations when applied to neuronal populations, as it can be physically harsh on fragile cell types (Binek et al. 2019) and is generally unsuitable for frozen tissues. A modification to FACS by alternatively sorting nuclei (FANS) works around this issue (Mussa et al. 2021), with other benefits such as avoiding dissociation‐induced gene expression changes (van den Brink et al. 2017) and improving the identification of rare cell types (Koenitzer et al. 2020; Santo et al. 2025). However, exclusion of the extranuclear space can result in loss of any fluorescent molecules expressed cytoplasmically; thus, one is limited to dyes tethered to the nuclear membrane, such as the SUN1‐GFP mouse line (Fernandez‐Albert et al. 2019), or GFP‐KASH transgenes (Swiech et al. 2015). Alternatively to bulk RNA sequencing, with or without sorting, greater acuity and insights into population heterogeneity can be provided by single‐cell RNA sequencing (scRNAseq; Lacar et al. 2016; Santoni et al. 2024), which is particularly useful for investigating molecular changes before memory allocation has occurred (i.e., when engram tagging systems cannot be used).

A subsequent level of investigation concerns the epigenome. Here, a first glimpse into epigenetic dynamics can be gained by measuring chromatin accessibility changes, which have been studied in engram cells at bulk (Marco et al. 2020) or single‐cell resolution (Santoni et al. 2024). When combined with scRNA seq in a multi‐omic sequencing experiment, this approach offers simultaneous insight into epigenetic‐transcriptional dynamics underlying memory allocation. DNA methylation, in turn, has been studied by bisulfite sequencing to determine differentially methylated sites on the DNA and how this governs excitability and thus allocation, while single nuclei approaches such as single‐nucleus methylome sequencing (snmC‐seq2; Luo et al. 2018) or multi‐omic sequencing (sn‐m3C‐seq; Lee et al. 2019; Liu et al. 2023) have yet to see the light of day in allocation studies.

Concerning protein interactions with chromatin, little research exists in the allocation field. A particularly useful technique for investigating both epigenetic marks and the occupancy of their writers/erasers is chromatin immunoprecipitation (ChIP; Tsankova et al. 2006). Interrogation of the DNA can be carried out either using quantitative polymerase chain reaction (ChIP‐qPCR) for a genomic region of interest (Marco et al. 2020) or next‐generation sequencing (ChIP‐seq) for an unbiased map of protein‐chromatin interactions (Johnson et al. 2007). For example, ChIP‐seq has been used in cultured neurons to identify CREB enrichment on the genome (Lesiak et al. 2013). ChIP‐seq can further be used to interrogate not only chromatin binding patterns but also the extent of post‐translational histone modifications (O'Geen et al. 2011), a clearly warranted goal given the implication of histone acetylation (Santoni et al. 2024) and methylation in memory allocation (Guo et al. 2024).

Despite the strengths of ChIP‐seq, its application to engram research is limited because of its requirement for a large cell number as input material (Gilfillan et al. 2012). The development of newer techniques such as CUT&RUN (Cleavage Under Targets and Release Using Nuclease; Skene and Henikoff 2017) and CUT&Tag (Cleavage Under Targets and Tagmentation; Kaya‐Okur et al. 2019) has been able to reduce this limiting factor, although they have yet to be applied to allocation research. CUT&Tag is particularly promising in this regard, as it has already been used at single cell resolution (Bartosovic et al. 2021; Bartosovic and Castelo‐Branco 2023; Kaya‐Okur et al. 2019) and can even encompass the profiling of several epigenetic marks on the same samples (Liu et al. 2025; Liu and He 2025; Wang et al. 2019).

Beyond protein and histone interactions with the chromatin, Hi‐C allows for the investigation of general chromatin architecture. Hi‐C involves formaldehyde‐driven cross‐linking on covalently bonded DNA to capture its physical contacts, which are then read by deep sequencing to map areas of the genome which are in contact with each other (Belton et al. 2012). This yields additional information on the three‐dimensional architecture of the chromatin at the time of allocation, and has already been applied at other stages of memory post‐encoding (Fernandez‐Albert et al. 2019; Marco et al. 2020). More recent techniques such as high‐resolution chromosome‐conformation‐capture carbon‐copy sequencing (5C‐seq) have provided more opportunities for higher resolution chromatin contact maps around genomic loci of interest Ferraiuolo et al. (2012). However, this tool has only been applied in neuronal cultures so far (Beagan et al. 2020). A caveat of such sequencing methods lies in inherent technical limitations resulting in biases with, for example, restriction site density and GC content. Additionally, these methods do not efficiently capture nuclear organisation in regards to proximity to the nuclear periphery. A technique known as genome architecture mapping (GAM) overcomes these limitations by taking cryopreserved and ‐sectioned tissue, microdissecting the nuclei and analysing the genomic material from that slice. If two chromatin regions are close in three‐dimensional space, they are more likely to be detected in the same slice. With random sampling and sectioning of the nuclei, a matrix is created which allows proximity inference of two or more regions in the three‐dimensional nuclear space (Beagrie et al. 2017). Additionally, there are techniques that allow for the visualisation of chromatin architecture at single‐cell resolution via fluorescence. For example, optical reconstruction of chromatin architecture (ORCA) takes advantage of array‐derived oligonucleotide probes (oligopaints) building on innovations in RNA and DNA fluorescence in situ hybridisation (FISH; Mateo et al. 2019). This allows for chromatin mapping in situ providing a valuable complement to sequencing‐based techniques. As of today, though, these techniques—as well as approaches to functionally manipulate the 3D chromatin architecture such as CLOuD9 (Morgan et al. 2017), CRISPR GO (Wang et al. 2018) and light activated dynamic looping (LADL; Kim et al. 2019)—have yet to be applied to the study of memory allocation.

Lastly, any epigenetic modification can also be studied through the modulation of the enzymes responsible for the epigenetic modifications. This can be achieved by traditional knock down or overexpression approaches in defined allocation‐relevant cell populations (Odell et al. 2020; Guo et al. 2024; Santoni et al. 2024) or by using CRISPR‐dCas9 epigenetic editing approaches (Heidenreich and Zhang 2015; Hamilton et al. 2018). These tools, when fused to an epigenetic enzyme such as CBP (Coda et al. 2025; Watt et al. 2025), allow for engram‐specific, locus‐resolved, time‐specific overexpression of particular epigenetic players, thereby enabling the functional interrogation of cell populations and of specific loci for memory formation.

## 6 Outlook

It is clear that, despite significant advances in the field of memory allocation and the application of various techniques, the investigative toolkit continues to and needs to evolve to open the door for future discoveries. For example, is there a way to directly bridge excitability and chromatin changes within the same cell? Hypothetically, this could be achieved by Patch‐seq modified to include ATAC‐seq or CUT&Tag‐style techniques. Patch‐seq allows for the profiling of both IE as well as gene expression changes by combining the already existing patch‐clamp technique with RNA sequencing (Cadwell et al. 2016; Rao‐Ruiz et al. 2019). If this labour‐intensive technique could be adapted for chromatin profiling, great insights into the link between IE and chromatin changes that underlie allocation at single cell resolution would be guaranteed.

Another unexplored dimension in memory allocation is a larger systems neuroscience level approach. One recent study has started to address this by combining CREB‐overexpression approaches with retrograde tracing to demonstrate an interconnected network where the allocation of memories in one brain region has a direct effect on the allocation of neurons in another (Lavi et al. 2023). This technique was termed CRANE (Cross‐Regional Afferent Network of Memory Ensembles) and was used to interrogate the connections between the LA and the insular cortex. Understanding a systems‐wide allocation network and how manipulations in one area affect others (which could be even further interrogated by measuring molecular repercussions not just in the manipulated cells but in other brain regions) could greatly improve the knowledge in the field. In particular, such approaches could clarify whether allocation is a local phenomenon or relies on the orchestrated activity of distributed processes throughout the brain.

Ultimately, advancing the field of memory allocation will require a shift towards integrative frameworks embracing its multidimensional nature. The challenge ahead therefore lies not only in linking excitability to chromatin states or mapping networks of interacting ensembles, but also situating these processes within the broader physiological states that govern the brain. Metabolic state, hormonal signalling, sleep, and even gut‐brain axis communication may all converge on the molecular and circuit mechanisms that bias the formation of an engram ensemble. Likewise, the contributions of astrocytes, inhibitory interneurons, and other non‐neuronal partners remain relatively unknown, yet are essential for a deeper understanding for memory allocation. Furthermore, considering the centrality of memory formation to cognition, an intriguing yet largely untested possibility is that certain cognitive dysfunctions may, at least in part, stem from the misallocation of memories. By weaving together molecular, cellular, systems, and organismal perspectives—and examining how these processes become perturbed in pathology—we may ultimately arrive at a unifying framework in which memory allocation is seen not as a purely neural event, but as an emergent property of the brain in constant dialogue with the body and its environment.

## Author Contributions

**Isabella Tarulli:** writing – original draft, conceptualization. **Rebecca Toscano‐Rivalta:** writing – original draft, conceptualization. **Lisa Watt:** writing – original draft, conceptualization. **Johannes Gräff:** writing – review and editing, funding acquisition, conceptualization.

## Funding

This work was supported by Stiftung Synapsis ‐ Alzheimer Forschung Schweiz AFS. Vallee Foundation. European Research Council, CoG 101043457. Chan Zuckerberg Initiative. Schweizerischer Nationalfonds zur Förderung der Wissenschaftlichen Forschung, 310030_197752, 310030_219342.

## Conflicts of Interest

The authors declare no conflicts of interest.

## Data Availability

Data sharing not applicable to this article as no datasets were generated or analysed during the current study.
